# High-Sensitivity C-Reactive Protein: A Potential Ancillary Biomarker for Malaria Diagnosis and Morbidity

**DOI:** 10.1155/2019/1408031

**Published:** 2019-04-04

**Authors:** Otchere Addai-Mensah, Max Efui Annani-Akollor, Linda Ahenkorah Fondjo, Enoch Odame Anto, Daniel Gyamfi, Lorraine Sallah, Dennis Agama, Richard Djabatey, Eddie-Williams Owiredu

**Affiliations:** ^1^Department of Medical Laboratory Technology, Faculty of Allied Health Sciences, College of Health Sciences, Kwame Nkrumah University of Science and Technology, Kumasi, Ghana; ^2^Department of Molecular Medicine, School of Medical Sciences, College of Health Sciences, Kwame Nkrumah University of Science and Technology, Kumasi, Ghana; ^3^Department of Physiology, School of Medical Sciences, College of Health Sciences, Kwame Nkrumah University of Science and Technology, Kumasi, Ghana

## Abstract

**Background:**

Malaria remains an important cause of morbidity and mortality in Africa. Previous studies that assessed C-reactive protein (CRP) have centered on the conventional method. This study evaluated the usefulness of high-sensitivity CRP (hs-CRP) in malaria diagnosis and morbidity in a pediatric population in Ghana.

**Methodology:**

A total of 267 subjects (100 microscopically proven nonmalarial parasitaemics as controls and 167 plasmodium parasitaemic subjects as cases), between the ages of 7 months and 18 years, were recruited for this case-control study. Blood samples were collected for malaria parasite density by microscopic examination; full blood count, electrolytes, and liver function tests using an automated analyzer; and hs-CRP levels by sandwich ELISA method.

**Results:**

The median hs-CRP concentration was lowest in the control group and increased significantly from low to high parasitaemia. The median hs-CRP level was significantly higher in high malaria parasitaemia compared to moderate and low malaria parasitaemia. Increasing hs-CRP cutoff (3.12-4.64 mg/L) presented with increasing specificity (79.3-93.1%) and sensitivity (96.4%-97.4%), except for moderate parasitaemia where a decline in sensitivity (80.9%) was observed. However, hs-CRP had relatively lower PPV but high NPV at low parasitaemia while both the PPV and NPV were moderate in moderate parasitaemia.

**Conclusion:**

hs-CRP yielded a high sensitivity, specificity, and accuracy for low, moderate, and high-grade malaria, respectively, and thus may serve as an effective supplementary diagnostic and prognostic biomarker for Plasmodium parasite infection. However, hs-CRP might not be readily useful yet for diagnostic purposes in hospitals due to the relatively low PPV and NPV for low and moderate parasitaemia and thus necessitates further studies in larger cohorts.

## 1. Introduction

Malaria is a widespread disease in the tropical and subtropical regions, affecting mostly sub-Saharan Africa, Asia, and Latin America [1]. As an important cause of morbidity and mortality in Africa, malaria exacts an enormous toll on life and property, with annual infection and mortality rates of 188 million and 395,000 individuals, respectively, with children below 5 years being the predominantly affected group [2]. Most of these children live within underdeveloped regions and depend on poorly resourced healthcare facilities, where laboratory personnel and investigations are inadequate. In Ghana, malaria remains the major cause of loss of days of healthy life, accounting for at least twenty percent (20%) of child deaths, forty percent (40%) of child hospital admissions, and over fifty percent (50%) of outpatient attendances [3–6].

In endemic areas, there may be afebrile presentation of malaria even in patients with high parasite density [7]. Confoundingly, the presence of fever, a classical symptom of malaria, could be due to other infections of viral or bacterial origin, which may be misconstrued as malaria. Moreover, other symptoms of malaria including headache, fatigue, joint aches, and abdominal discomfort may also occur in viral or bacterial infections [8]. These pose difficulties in the identification of patients with or without malaria at presentation. Due to some overlaps in the presentation of malaria and other viral and bacterial infections, especially in endemic countries, the WHO recommends that microscopy or rapid diagnostic test (RDT) is a requirement for malaria diagnosis before treatment in suspected malaria cases [8]. However, in the event of unavailability of such methods, other biomarkers could be of expediency.

As a result, some blood tests, including leukocyte count and liver function tests, have previously been used to assess the malaria severity but have shown to have a rather low sensitivity. Other markers related to immune activation in malaria have also been explored. C-reactive protein (CRP), an acute phase reactant, whose plasma concentration increases during inflammatory disorders [9], has gained considerable attention as a biomarker in malaria [10]. This is very important in endemic regions where conventional disease manifestations like fever may be absent even in patients with high parasite load [10]. CRP levels have been shown to correlate strongly with blood parasite density in patients with or without clinical symptoms [10, 11]. Its levels have been associated with malaria [12, 13] as well as other infections [14, 15], and it plays a pivotal role in the activation of platelet and the complement pathway [16] in addition to binding to infected erythrocytes and helping in their clearance [17].

Despite the reported usefulness of traditional CRP tests in malaria, a more sensitive approach could potentially enhance early detection and monitoring of malaria severity. High-sensitivity CRP (hs-CRP) allows the detection of low-range differences in C-reactive protein levels (1-10 mg/L) as opposed to the conventional method (10-1000 mg/L). Previous studies have focused on its use in cardiovascular diseases [18–21], sepsis [22], HIV [23], and cognitive impairment [24], but there is currently no study on malaria and its associated morbidity. Additionally, with evidence of the effect of ethnic and geographic variations on the physiological processes in the body, it is essential that geography-specific cutoff values for these markers be assessed to ensure diagnostic accuracy and effective clinical decision-making in this era of evidence-based medicine. This study, therefore, sought to evaluate hs-CRP and determine its diagnostic applicability as a marker for malaria in a pediatric population in Ghana.

## 2. Materials and Methods

### 2.1. Study Design/Study Area

This case-control study was conducted at Komfo Anokye Teaching Hospital (KATH) located in Kumasi, the capital of the Ashanti Region in Ghana, between August 2015 and April 2016. Kumasi is Ghana's second largest city located about 300 km from the national capital, Accra. The city of Kumasi lies between latitude 6.35 °N and 6.40 °N and longitude 1.3 °W and 1.35 °W, and it is 150 km^2^ in size and is bounded by four districts and is located in the rainforest zone of West Africa with a population of about 2 million inhabitants [25]. The climatic conditions of Kumasi are typical of that of a tropical region and therefore aid the transmission of malaria parasites [26].

### 2.2. Inclusion and Exclusion Criteria

Participants between the ages of 7 months to 18 years were recruited for the study. Participants positive for malaria were recruited as cases (children presenting at hospital with malaria) while those negative for malaria were considered as controls. The study participants were obtained through random sampling technique. Patients' corresponding clinical data was retrieved from the hospital's archive. Subjects with evidence of recent chronic illnesses, chronic liver disease, inflammatory diseases, and viral exanthema as well as those receiving anti-inflammatory drugs were excluded.

### 2.3. Ethical Considerations

Ethical approval for this study was obtained from the Committee on Human Research Publication and Ethics (CHRPE) of the School of Medical Sciences, Kwame Nkrumah University of Science and Technology (CHRPE/AP/184/16), and also from the Research and Development Department of KATH. Written informed consent was obtained from parents or guardians after the aim and objectives of the study had been explained to them.

### 2.4. Sample Collection and Preparation

Five (5) milliliters of blood was aseptically collected from the antecubital vein of each respondent at the hospital. Blood samples were collected immediately after written informed consent was received. Three (3) milliliters of whole blood was dispensed into K_3_ EDTA tubes and mixed to prevent clotting. The whole blood in the EDTA tube was used for plasmodium parasite identification and parasite density as well as full blood count (FBC). The remaining 2 mL was dispensed into gel separator tubes, placed in a centrifuge, and spun at 3000 rpm for 10 minutes to obtain the serum. The serum was stored at -20°C until analysis. Serum was used for the estimation of hs-CRP, liver function tests, and electrolyte.

### 2.5. Parasite Density and Haematological and Biochemical Assays

Standard thick and thin films were prepared on clean grease free slides for each participant and the thin films fixed with absolute methanol, followed by staining with 10% Giemsa and subsequent microscopic examination for malaria parasite [26]. Using the absolute WBC obtained from the FBC of each participant using the Sysmex KX 4000i haematology analyzer (Sysmex Corporation, Kobe, Japan), the number of malaria parasites per microliter of blood was calculated as follows: (number of parasites counted/WBC counted)×WBC count/*μ*l of participant. Blood films were declared negative if no parasites were seen in 200 oil-immersion fields as described by Squire et al. [27]. For *P. falciparum*counts ≥ 100 parasites at a high-power field in thick smear, parasite counts were confirmed in thin films (against 2,000 red blood cells) and recalculated with 200 WBCs. Malaria parasitaemia was graded as low parasitaemia (≥1 to <10,000 parasites/*μ*L), moderate parasitaemia (≥10,000 to <100,000 parasites/*μ*L), and high parasitaemia (≥100, 000 parasites/*μ*L) according to WHO criteria [28].

Serum bilirubin, alanine aminotransferase (ALT), aspartate aminotransferase (AST), creatinine, sodium, potassium, chloride, and calcium estimation were assayed using the Cobas Integra automated chemistry analyzer (Roche Cobas Integra 400 Plus, Roche Diagnostics, USA). The reagent for the determination of hs-CRP was purchased from DRG International Inc. (EIA-3954, DRG International Inc., Springfield Township, USA) and analyzed based on the principle of solid phase sandwich enzyme-linked immunosorbent assay according to the manufacturer's instructions (standardized with an intra-assay CVs = 2.5%-4.1% and interassay CVs = 2.3%-7.5%). Briefly, 10 *μ*L of hs-CRP standards, diluted specimens, and controls (100-fold dilution) was dispensed into appropriate precoated microtitre wells followed by 100 *μ*L of hs-CRP enzyme conjugate reagent, mixed thoroughly for 30 seconds, and incubated at room temperature for 45 minutes. After, the incubation mixture was removed from the wells by flicking plate contents into a waste container, rinsed, and flicked again with deionized water. Residual water droplets were removed by sharply striking the wells onto absorbent paper. 100 *μ*L of tetramethylbenzidine (TMB) solution was then dispensed into each well and mixed gently for 5 seconds, followed by 20 minutes' incubation at room temperature. The reaction was stopped by adding 100 *μ*L of stop solution to each well and gently mixed for 30 seconds. The absorbance of the final colored product was measured spectrophotometrically at 450 nm using the Thermo Electron Multiskan EX plate reader (Shanghai, China). The mean absorbance value (OD_450_) for each set of reference standards, controls, and samples were calculated. The calculated mean OD_450_ obtained for each reference standard was used to construct a standard curve and the concentrations of samples and controls determined from the standard curve. The obtained values of the patient samples and control sera were then multiplied by the dilution factor of 100 to obtain actual hs-CRP concentration. Patient samples with hs-CRP concentrations greater than 10 mg/L were further diluted 10-fold after the initial 100-fold dilution (total dilution 1 : 1,000), and the final CRP values were multiplied by 1,000 to obtain the actual concentration of hs-CRP.

Daily calibration and maintenance of the analyzer was performed according to the manufacturer's instructions as previously described [29]. Briefly, internal quality control (QC) and precision of analysis was assessed by internal quality control and accuracy was determined based on external quality control performance (United Kingdom International External Quality Assessment Scheme (UK IEQAS)). Uniformity of calibration was also ensured.

### 2.6. Definition of Terms

Anaemia was defined as haemoglobin level < 13.5 g/dL for males and < 12.0 g/dL for females. Thrombocytopaenia and leukocytosis was defined by platelet count < 150.0 × 10^3^/*μ*L and WBC > 4.5 × 10^3^/*μ*L of whole blood, respectively.

### 2.7. Statistical Analysis

All categorical data were presented as frequency (percentages), and the Chi-square test statistics was used to test for association between variables. The Mantel-Haenszel test of trend was used to examine rates across levels of parasitaemia. The trend across age ranges was evaluated by the Jonckheere trend test followed by Kendall's tau-*b* to estimate the effect size. Continuous variables were presented as mean ± standard deviation for parametric variables and median (interquartile range) for nonparametric variables. The Kruskal-Wallis test was used to assess significance of differences for nonparametric variables between controls and different grades of malaria parasitaemia, followed by the Bonferroni post hoc multiple comparison test. One-way ANOVA was used to test for significance of differences for parametric variables between controls and different grades of malaria parasitaemia, followed by the Tukey post hoc multiple comparison test. Partial correlation was used to test for associations between various parameters upon adjusting for age and gender. The ROC analysis was used to ascertain the diagnostic performance of hs-CRP for malaria. Confidence was set at 95%, and *p* value less than 0.05 was considered statistically significant. All statistical analyses were performed using IBM SPSS 20.0 Statistics and GraphPad Prism 7 version 7.04.

## 3. Results

A total of 267 participants comprising 167 confirmed malaria positive cases (85 females and 82 males) and 100 malaria-negative controls (30 males and 70 females) were recruited for the study.

The prevalence of low, moderate, and high-grade malaria parasitaemia were 16.8%, 37.1%, and 46.1%, respectively. A higher proportion of the participants with low, moderate, and high parasitaemia were 1-5 years old, in elementary school, used ITN, were anaemic, and had normal platelet and WBC counts. Most of the participants with moderate and high parasitaemia were females, and there was an equal proportion of males and females with low parasitaemia (Table 1).

**Figure 1:**
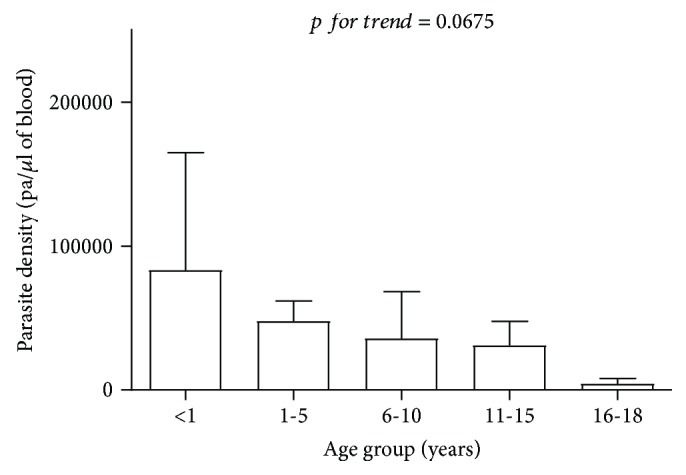
Parasite density of study cases stratified by age groups.

As shown in Figure 1, malaria parasite density decreased across increasing age groups.

**Figure 2:**
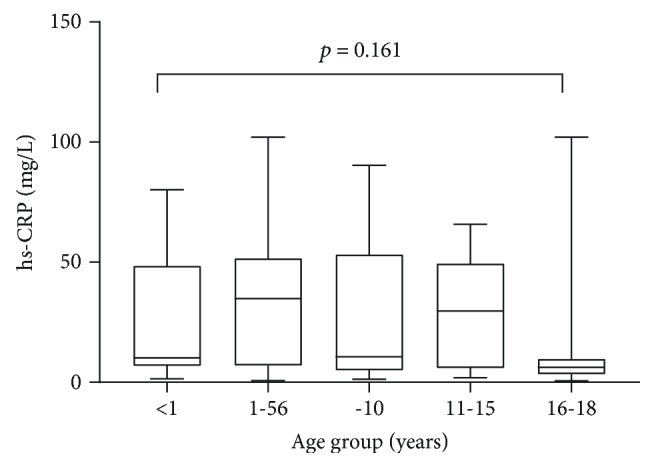
Levels of hs-CRP by age ranges.

Figure 2 shows hs-CRP levels across different age groups. There was no statistically significant difference between the levels of hs-CRP across the age ranges.

**Figure 3:**
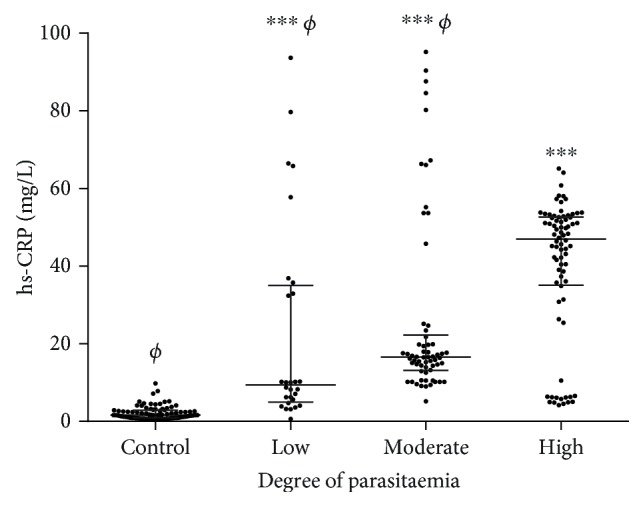
Levels of hs-CRP among different grades of malaria parasitaemia.

Our results show that the median hs-CRP concentration is lowest in the control group and increases significantly from low to high parasitaemia (Figure 3).

As shown in Table 2 and Figure S1, increasing hs-CRP cutoff values (3.12-4.64 mg/L) presented with increasing specificity (79.3-93.1%), sensitivity (96.4%-97.4%), and excellent AUCs, except for moderate parasitaemia where a decline in sensitivity (80.9%) was observed. However, there were variabilities in the predictive values. hs-CRP had a relatively low PPV but high NPV at low parasitaemia while both the PPV and NPV were moderate in moderate parasitaemia.

## 4. Discussion

Malaria is a disease of global health importance requiring urgent interventions to reduce the disease burden. The WHO has proposed appropriate and early diagnosis as a way to decrease morbidity and mortality. Some biomarkers have been assessed in this effect. A significant one is C-reactive protein. Previous studies on usefulness of C-reactive protein in malaria have only centered on the conventional CRP [11, 12, 30–34] while those involving hs-CRP have focused on cardiovascular diseases [18–21], sepsis [22], HIV [23], and cognitive impairment [24]. However, till date, no study has been conducted to assess the expediency of hs-CRP in malaria and its attendant morbidity. This study, to the best of our knowledge, is the first study to evaluate the levels and usefulness of hs-CRP as a marker for malaria diagnosis and morbidity in a pediatric cohort in Ghana.

This study reports increasing level of hs-CRP across increasing parasite density among children with malaria. Coupled with the high sensitivity and specificity, this finding suggests that the cutoff values of hs-CRP obtained in this study may serve as a potential marker for malaria morbidity and help to identify patients with a chronic low-grade proinflammatory state, a critical contributor to the clinical manifestations of malaria [35, 36]. Additionally, with evidence of the effect of ethnic and geographic variations on the physiological processes in the body, it is imperative that geography-specific cutoff values for these markers be assessed to ensure diagnostic accuracy and effective clinical decision-making in this era of evidence-based medicine. Nonetheless, the WHO recommends that microscopy or rapid diagnostic test (RDT) is a requirement for malaria diagnosis in suspected malaria cases [8] and hs-CRP must therefore not be considered as a substitute for appropriate parasitological diagnosis. Additionally, the high negative predictive values obtained for hs-CRP in this study imply that there is a high possibility that patients with normal/reduced hs-CRP levels have no malaria parasitaemia. As such, normal hs-CRP levels may be used in excluding malaria in febrile children in the event where malaria microscopy and RDT may not be readily available, a commonly encountered obstacle in Africa. Moreover, the RDTs employed in most parts of Africa for the diagnosis and the monitoring of treatment for malaria infection depend on the histidine-rich protein 2 (HRP-2) [37]. However, the WHO reports that HRP-2 may present false-negative results due to deletion of the *pfhrp 2 gene* [38], making diagnosis and monitoring of therapy difficult. Measurement of hs-CRP may thus be useful as a supplement to these tests to ensure definitive diagnosis and informed decision-making.

Nevertheless, the relatively low PPV for low parasitaemia and the moderate PPV and NPV in moderate parasitaemia suggest that, though hs-CRP may assist in making some informed clinical decisions, especially in highly malaria-endemic areas, where fever is frequently equivalent to malaria, it might not be readily useful yet for diagnostic purposes in hospitals and thus necessitates further studies in larger cohorts.

Additionally, studies have shown that, in malaria-endemic regions, children acquire passive immunity from their mothers. However, this immunity is limited to the first 6 months of life, after which the immunity is lost [39, 40], and children develop their own immunity to malaria through exposure [39, 40]. These reasons, hinged with knowledge of prophylaxis and the use of insecticide-treated nets, may make older children less prone to high malaria parasite burden as observed in this study. However, there were no statistically significant differences between the levels of hs-CRP across the age ranges.

This study is somewhat limited by the large age range of the cohort and the fact that only microscopy was utilized for malaria diagnosis and not tests such as RDTs or the more sensitive diagnostic tools such as PCR. Additionally, the increased hs-CRP level could be due to other inflammatory conditions such as viral and bacterial infections which were not screened for.

## 5. Conclusion

hs-CRP yielded a high sensitivity, specificity, and accuracy for low, moderate, and high grade malaria, respectively, and thus may serve as an effective supplementary diagnostic and prognostic biomarker for Plasmodium parasite infection. However, hs-CRP might not be readily useful yet for diagnostic purposes in hospitals due to the relatively low PPV and NPV for low and moderate parasitaemia and thus requires further studies in larger cohorts.

## Figures and Tables

**Table 1:** Sociodemographic characteristics of study participants.

Characteristics	Control (*N* = 100)	Low parasitaemia (*N* = 28; 16.8%)	Moderate parasitaemia (*N* = 62; 37.1%)	High parasitaemia (*N* = 77; 46.1%)	Total (*N* = 167; 100%)	*p* value for trend
*Age group (years)*						**<** *0.0001*
<1	0 (0.0%)	3 (10.7%)	4 (6.4%)	5 (6.5%)	12 (7.1%)	
1-5	1 (1.0%)	15 (53.6%)	33 (53.2%)	50 (64.9%)	98 (58.6%)	
6-10	35 (35.0%)	3 (10.7%)	10 (16.1%)	8 (10.4%)	21 (12.6%)	
11-15	62 (62.0%)	2 (7.1%)	10 (16.1%)	12 (15.6%)	24 (14.4%)	
16-18	2 (2.0%)	5 (17.9%)	5 (8.1%)	2 (2.6%)	12 (7.2%)	
*Gender*						*0.0281*
Male	30 (30.0%)	14 (50.0%)	30 (48.4%)	38 (49.5%)	82 (49.1%)	
Female	70 (70.0%)	14 (50.0%)	32 (51.6%)	39 (50.5%)	85 (50.9%)	
*Level of education*						**<** *0.0001*
No education	0 (0.0)	9 (32.1%)	24 (38.7%)	30 (39.9%)	63 (37.7%)	
Elementary	80 (80%)	15 (53.6%)	27 (43.5%)	34 (44.2%)	76 (45.5%)	
JHS	18 (18%)	1 (3.6%)	7 (11.0%)	6 (7.8%)	14 (8.4%)	
SHS	0 (0.0%)	3 (10.7%)	4 (6.5%)	7 (9.1%)	14 (8.4%)	
*ITN*						*0.0007*
Yes	41 (41.0%)	19 (67.9%)	41 (66.1%)	52 (67.5%)	112 (67.1%)	
No	59 (59.0%)	9 (32.1%)	21 (33.9%)	25 (32.5%)	55 (32.9%)	
*Haemoglobin status*						**<** *0.0001*
Anemic	36 (36.0%)	20 (71.4%)	50 (80.6%)	69 (89.6%)	139 (83.2%)	
Nonanemic	64 (64.0%)	8 (28.6%)	12 (19.4%)	8 (10.4%)	28 (16.7%)	
*Platelet count*						*0.0009*
Thrombocytopenia	1 (1.0%)	2 (7.1%)	12 (19.4%)	12 (15.6%)	26 (15.7%)	
Normal level	99 (99.0%)	26 (92.9%)	50 (80.6%)	65 (84.4%)	141 (84.4%)	
*WBC count*						**<** *0.0001*
Leukocytosis	2 (2.0%)	2 (7.1%)	11 (17.7%)	23 (29.9%)	36 (21.6%)	
Normal	98 (98.0%)	26 (92.9%)	51 (82.3%)	54 (70.1%)	131 (78.4%)	

Mantel-Haenszel test of trend was used to examine rates across levels of parasitaemia. *p* < 0.05 was considered statistically significant (*p* values of significant variables are in bold print). ITN: insecticide-treated net.

**Table 2:** Diagnostic performance of CRP in relation to the degree of parasitaemia.

Parasitaemia	hs-CRP (mg/L) cutoff	Sensitivity (95% CI)	Specificity (95% CI)	PPV	NPV	TP	TN	FP	FN	Accuracy	AUC (95% CI)
Low	3.1176	*96.4* (80.5-100.0)	*79.3* (69.5-86.5)	60.0	98.6	27	69	18	1	0.8348	0.93 (0.86-0.99)
Moderate	4.5398	*80.9* (69.4-88.8)	*91.9* (83.9-96.2)	87.9	86.9	51	80	7	12	0.8733	0.89 (0.84-0.95)
High	4.6418	*97.4* (90.2-99.8)	*93.1* (85.4-97.0)	92.5	97.6	74	81	6	2	0.9509	0.99 (0.99-0.99)

## Data Availability

The data used to support the findings of this study are included within the article.

## Supplementary Materials

Supplementary Materials Table S1: partial correlation between hs-CRP and haematological and biochemical parameters after adjusting for age and gender among children with malaria. Table S2: haematological and biochemical parameters categorized by degree of malaria parasitaemia. Figure S1: AUCs for hs-CRP in detection of low parasitaemia (A), moderate parasitaemia (B), and high parasitaemia (C).
