# Supplementary material for: High-Sensitivity C-Reactive Protein: A Potential Ancillary Biomarker for Malaria Diagnosis and Morbidity
Source: Dis Markers. 2019 Apr 4;2019:1408031. doi: 10.1155/2019/1408031 (PMC6476067; doi:10.1155/2019/1408031)
Supplement: Supplementary Materials — Table S1: partial correlation between hs-CRP and haematological and biochemical parameters after adjusting for age and gender among children with malaria. Table S2: haematological and biochemical parameters categorized by degree of malaria parasitaemia. Figure S1: AUCs for hs-CRP in detection of low parasitaemia (A), moderate parasitaemia (B), and high parasitaemia (C). [file 1408031.f1.pdf]

High sensitivity C-reactive protein: A potential ancillary biomarker for malaria diagnosis and morbidity

SUPPLEMENTARY DATA

Table S1 shows the partial correlation between hs-CRP and hematological and biochemical parameters after adjusting for age and gender among children with malaria. There were negligible correlations between hs-CRP and the haemato-biochemical parameters.

Table S1. Partial correlation between hs-CRP and hematological and biochemical parameters after adjusting for age and gender among children with malaria

Correlation	Correlation co-efficient
Hs-CRP vs. Na^+	-0.162
Hs-CRP vs. K^+	-0.089
Hs-CRP vs. Cl^-	-0.146
Hs-CRP vs. AST	-0.076
Hs-CRP vs. ALT	-0.026
Hs-CRP vs. AST/ALT	-0.088
Hs-CRP vs. Total Bilirubin	0.086
Hs-CRP vs. Creatinine	-0.045
Hs-CRP vs. Ca^{2+}	-0.049
Hs-CRP vs. Haemoglobin	-0.235
Hs-CRP vs. WBC	-0.033
Hs-CRP vs. RBC	-0.156
Hs-CRP vs. Platelet	-0.292
Hs-CRP vs. Neutrophil	0.161
Hs-CRP vs. Lymphocyte	-0.178
Hs-CRP vs. Mps	0.276

WBC; White blood cell, RBC; Red blood cell, Mps, Malaria parasite, $p < 0.05$ was considered statistically significant (p values of significant variables are in bold print).

Table S2 shows the hematological and biochemical parameters categorized by degree of malaria parasitaemia. Participants with high malaria parasitaemia had statistically significant elevated temperature, WBC count, Neutrophil count, AST, and Total bilirubin, with a corresponding lower

weight, haemoglobin level, RBC count, platelet count, lymphocyte count, sodium ion, chloride ion, and Creatinine level.

Table S2. Hematological and biochemical parameters categorized by degree of malaria parasitaemia

Parameters	Control (N=100)	Low (N=28)	Moderate (N=62)	High (N=77)	P-value	Significant pairs
Temperature (ºC)	38.02 ± 0.23	37.1 ± 0.21	37.87 ± 0.12	39.11 ± 0.34	< 0.0001	L vs H ; M vs H
Weight (kg)	36.09 ± 1.11	21.99 ± 3.19	22.53 ± 2.12	20.4 ± 1.72	< 0.0001	Ctrl vs L, M, and H
Haemoglobin (g/dl)	12.22 ± 0.08	10.54 ± 0.39	10.58 ± 0.52	9.97 ± 0.19	< 0.0001	Ctrl vs L, M, and H
WBC ($x10^3$/µl)	6.60 ± 0.25	7.25 ± 0.51	8.70 ± 0.47	9.17 ± 0.44	< 0.0001	Ctrl vs M, and H; L vs H
RBC ($x10^6$/µl)	4.82 ± 0.04	4.43 ± 0.17	4.19 ± 0.11	4.11 ± 0.08	< 0.0001	Ctrl vs M, and H
Platelet ($x10^9$/l)	336.6 ± 7.83	267.8 ± 16.07	240.6 ± 13.21	179.6 ± 7.46	< 0.0001	Ctrl vs L, M, and H; H vs L, and M
Neutrophil (%)	35.94 ± 1.01	55.37 ± 2.98	53.24 ± 2.26	63.96 ± 1.92	< 0.0001	Ctrl vs L, M, and H; H vs M
Lymphocyte (%)	50.21 ± 0.93	35.25 ± 3.42	37.79 ± 2.36	28.88 ± 1.84	< 0.0001	Ctrl vs L, M, and H ; H vs M
Na^+ (mmol/l)	139.1 ± 0.28	138.5 ± 1.42	138.9 ± 1.03	132.9 ± 2.08	0.0009	Ctrl vs H; H vs M
K+ (mmol/l)	4.01 ± 0.04	4.52 ± 0.13	4.41 ± 0.12	4.17 ± 0.10	0.001	Ctrl vs L, and M
Cl- (mmol/l)	103.2 ± 0.23	104.2 ± 1.12	104.1 ± 0.68	100.9 ± 1.23	0.0229	H vs M
AST (UI/L)	25.49 ± 0.65	35.65 ± 4.12	36.54 ± 2.19	39.81 ± 2.62	< 0.0001	Ctrl vs L, M, and H
ALT (UI/L)	24.77 ± 0.94	15.71 ± 2.78	15.71 ± 1.04	16.22 ± 1.23	< 0.0001	Ctrl vs L, M, and H
AST/ALT Ratio	1.19 ± 0.07	2.73 ± 0.27	2.70 ± 0.21	2.72 ± 0.13	< 0.0001	Ctrl vs L, M, and H
Total Bilirubin (mg/dL)	0.71 ± 0.05	0.59 ± 0.11	0.91 ± 0.16	1.19 ± 0.17	0.0043	Ctrl vs H
Creatinine (mg/dL)	0.72 ± 0.03	0.58 ± 0.06	0.52 ± 0.03	0.48 ± 0.03	< 0.0001	Ctrl vs M, and H
Total Ca^{2+} (mg/dL)	6.86 ± 0.13	9.61 ± 0.39	8.90 ± 0.23	8.89 ± 0.25	< 0.0001	Ctrl vs L, M, and H

One-way ANOVA was used to compare continuous variables among groups, followed by the Tukey's Honestly Significant Difference test for pairwise comparison. $p < 0.05$ was considered statistically significant (p values of significant variables are in bold print). RBC, Red blood cell, AST; Aspartate transaminase, ALT; Alanine transaminase, hs-CRP; high sensitivity C-reactive protein.

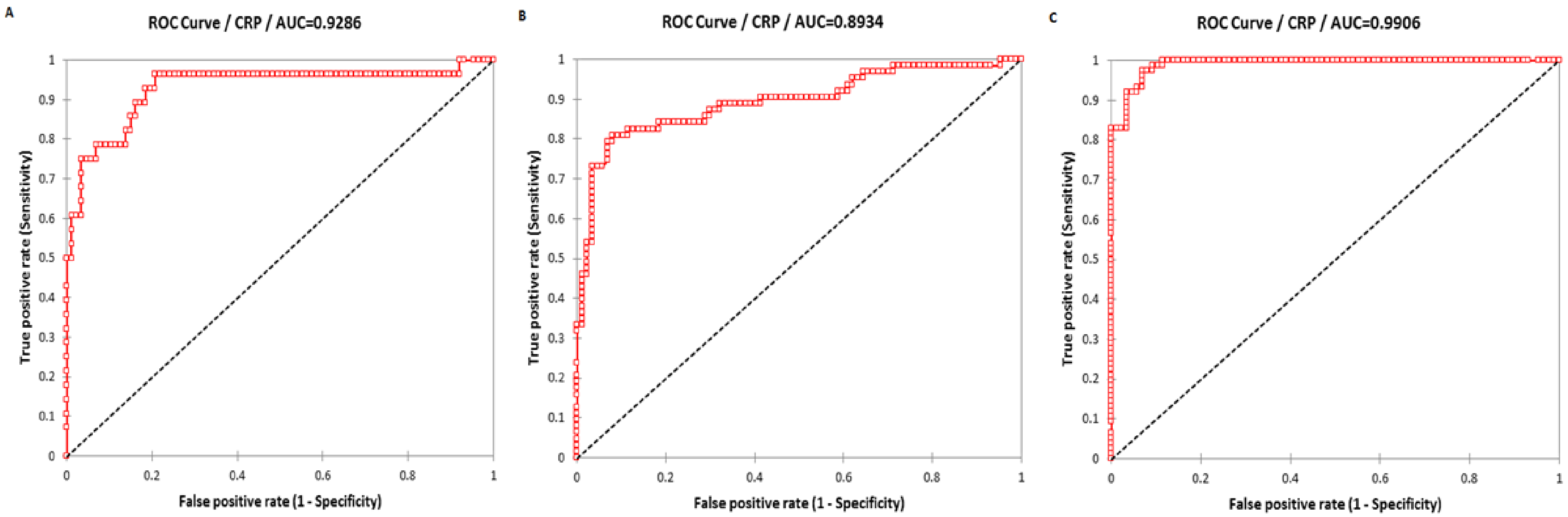

Figure S1. AUC for hs-CRP in detection of Low parasitaemia (A), Moderate parasitaemia (B) and High parasitaemia (C)
